# Plasma cell-free DNA measured prior to renal replacement therapy initiation is not associated with incident adverse outcomes, hospitalizations or malignancies in chronic kidney disease stage 4–5 patients

**DOI:** 10.1371/journal.pone.0353258

**Published:** 2026-07-07

**Authors:** Niilo Liuhto, Jenni Tuominen, Noora Manni, Markus Hakamäki, Roosa Lankinen, Tomi Toukola, Jonna Virtanen, Kaj Metsärinne, Mikko J. Järvisalo, Tapio Hellman

**Affiliations:** 1 Kidney Centre, Turku University Hospital and University of Turku, Turku, Finland; 2 Department of Genomics, Turku University Hospital and University of Turku, Turku, Finland; 3 Department of Nephrology, Vaasa Central Hospital, Vaasa, Finland; 4 Department of Internal Medicine, Wellbeing services county of Satakunta, Pori, Finland; Tokyo Medical University: Tokyo Ika Daigaku, JAPAN

## Abstract

Inflammation is an inherent feature of advanced chronic kidney disease (CKD) and associated with adverse outcomes. Several inflammatory biomarkers have been shown to be associated with mortality in CKD. Cell-free DNA (cfDNA) is a novel biomarker for inflammation which has not been previously examined in patients with CKD stage 4–5 not undergoing dialysis. cfDNA was extracted from plasma and quantified with Qubit Flex Fluorometer using dsDNA High Sensitivity kit in 138 patients with CKD stage 4–5 not undergoing dialysis at baseline and at a control time point of median 2.7 years of follow-up. A ratio of control and baseline measurement adjusted for an increment of one year of follow-up was calculated. Associations between cfDNA at baseline and all-cause mortality, major adverse cardiovascular and cerebrovascular events (MACCE, defined as a composite outcome of acute myocardial infarction, coronary revascularization, ischemic or hemorrhagic stroke and cardiovascular death), emergency room (ER) visits, hospitalizations or incident malignancies were assessed. Within a median follow-up of 6.2 years, no associations were observed between cfDNA and mortality, MACCEs, ER visits, hospitalizations or incident malignancies. cfDNA control measurement and cfDNA delta ratio was available in 101 patients. Patients who had received a kidney transplant by the control cfDNA measurement had significantly higher cfDNA delta ratio compared to patients not on renal replacement therapy (RRT) and those undergoing dialysis (p < 0.001 for both comparisons). Patients undergoing dialysis at the time of the control cfDNA measurement had higher cfDNA delta ratio (p = 0.003) compared to patients not on RRT. The present study is the first to show that cfDNA is not associated with adverse outcomes in patients with advanced CKD not undergoing dialysis at baseline. Furthermore, our results provide unique data on the evolution of cfDNA levels in predialysis CKD stage 4–5 patients transitioning to different modalities of RRT.

## Introduction

Chronic kidney disease (CKD) is associated with an elevated risk of mortality, which increases further with the progression of CKD. [1] Several cardiovascular and inflammatory biomarkers including albumin, interleukin-6, C-reactive protein (CRP), cardiac troponin and natriuretic peptides have been explored in previous literature, as well as, in our work and shown to be associated with mortality in patients with CKD stage 4–5. [2–6] However, few biomarkers are clinically applicable or used in contemporary clinical practice and, thus, new approaches are warranted.

Cell-free DNA (cfDNA) consists of extracellular DNA fragments of various sizes that circulate in the plasma. cfDNA is released into circulation from cells that have undergone apoptosis or necrosis or as a product of active DNA release mechanisms and thus thought to potentially serve as a biomarker of cellular death and tissue damage. [7] Elevated concentrations of plasma cfDNA have been observed in various pathological processes including inflammatory [8–9] and malignant diseases [10]. Furthermore, elevated cfDNA has been observed in hospitalized patients with traumatic injuries, sepsis or acute myocardial infarction. [11–13] Elevated cfDNA levels have also been associated with several traditional cardiovascular risk factors, such as hypertension, plasma lipids and inflammatory biomarkers. [14] Moreover, cfDNA has been suggested to serve as an independent predictor of mortality. [15] However, few data exist on the link between cfDNA and adverse outcomes in advanced CKD.

The aim of this study was to examine the association between cfDNA and long-term adverse outcomes including mortality, major adverse cardiovascular and cerebrovascular events (MACCE), emergency room (ER) visits, hospitalizations and incident malignancies in CKD stage 4–5 patients not undergoing dialysis at baseline. Furthermore, we sought to analyze the changes in cfDNA concentration as patients transit from the predialysis phase to maintenance dialysis or kidney transplantation (i.e., renal replacement therapy, RRT) or continue on conservative care.

## Materials and methods

### Study design

The Chronic Arterial Disease, Quality of Life and Mortality in Chronic Kidney Disease (CADKID, http://www.ClinicalTrials.gov, NCT04223726) is a prospective follow-up study that enrolled 210 participants between 22 August 2013 and 30 September 2017 at the predialysis outpatient clinic of Turku University Hospital Kidney Centre. All consented patients were above 18 years of age, had estimated glomerular filtration rate (eGFR) below 30 ml/min/m^2^ and were not receiving RRT at the time of recruitment.

**Fig 1:**
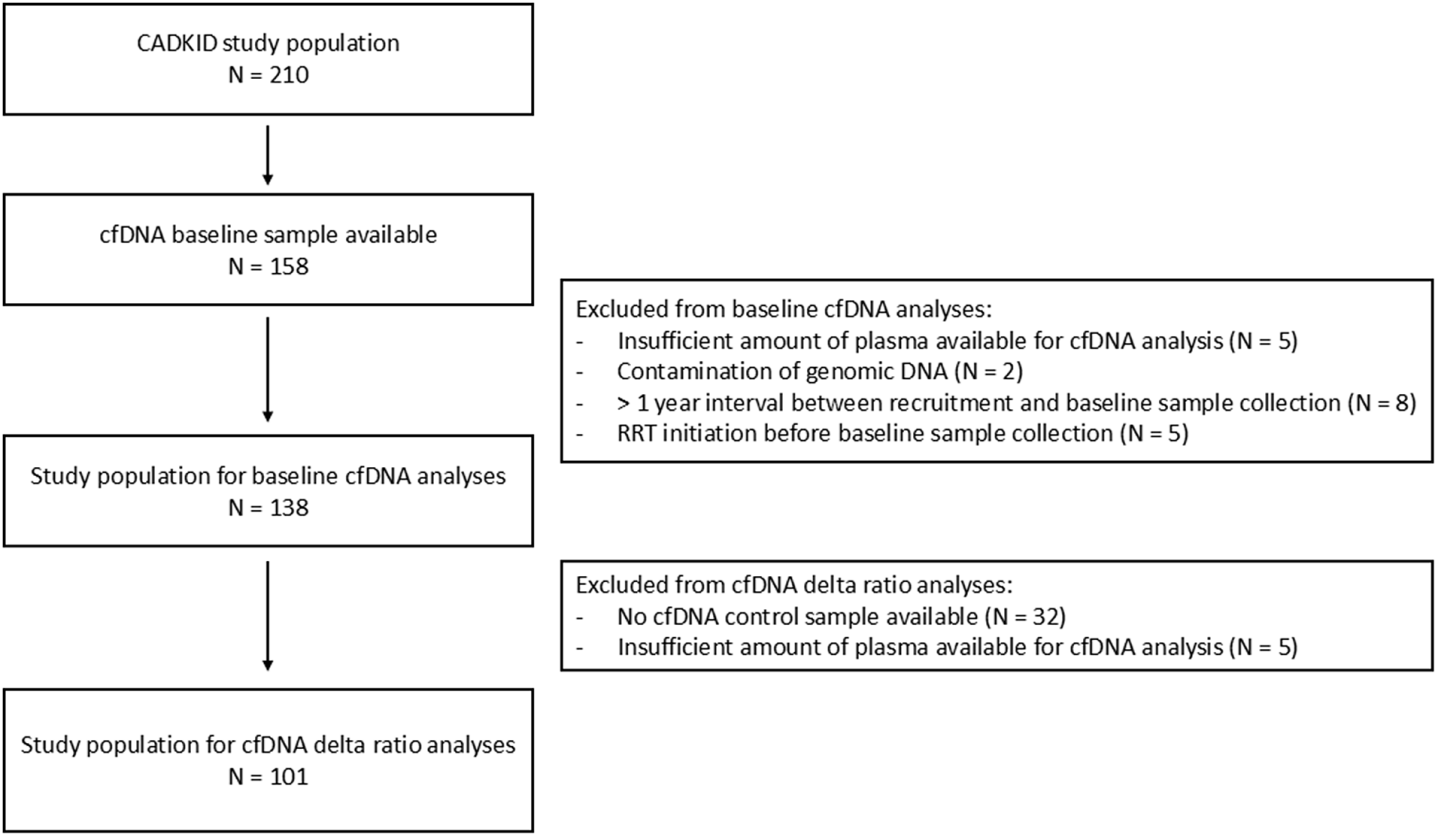
Flowchart of the study population.

Frozen plasma samples were collected after patient recruitment for predetermined cfDNA analyses. Patients were excluded if there was a delay of more than one year between recruitment and baseline cfDNA measurement, or if RRT had been initiated prior to the baseline cfDNA sample collection (Fig 1). 138 patients with baseline cfDNA measurements were included in the study, of whom 101 had a corresponding control/follow-up cfDNA sample.

The predetermined interval between baseline and control cfDNA measurement was set at 2 years but due to variation in the realized timing of the cfDNA control measurements, the cfDNA delta ratio was calculated as the ratio of control and baseline measurement adjusted for an increment of one year of follow-up.

### Outcomes and definitions

The outcome data from the electronic patient record system of Turku University Hospital used in all four hospitals of specialized care in South-West Finland were accessed for research purposes between 19/01/2024 and 30/04/2024 and the researchers manually collected the outcomes from recruitment until 31/12/2022. The measured study outcomes included all-cause mortality, MACCEs, ER visits, hospitalizations and incident malignancies. Hospitalization was defined as an episode of inpatient care in Turku University Hospital and included both elective and non-elective episodes. MACCE was defined as the composite outcome of acute myocardial infarction, coronary revascularization, ischemic or hemorrhagic stroke and cardiovascular death. Incident malignancies were denoted as any new solid organ or hematologic cancer (excluding localized non-melanoma malignancies of the skin) observed during follow-up. The total follow-up time started from the date of the baseline cfDNA sample and ended on 31/12/2022 or at the time of death. Furthermore, a shorter predialysis follow-up period censoring patients at the time of RRT initiation was used for additional outcome analyses. All laboratory measurements were performed in the Turku University Hospital Laboratories (Tyks Laboratories). eGFR calculation was performed using the CKD-EPI (Chronic Kidney Disease Epidemiology Collaboration) equation [16].

### cfDNA analysis

Blood samples were collected into EDTA tubes which were centrifuged, and the separated plasma was stored at −80 °C until analysis, in accordance with the clinical standards of the Tyks Laboratories (TYKSLAB). cfDNA was extracted from 1.5 ml of plasma using a QIAsymphony DSP Circulating DNA kit (QIAGEN, Hilden, Germany) according to the manufacturer’s instructions, and eluted in 60 µl of elution buffer. The fragment size distribution of the isolated cfDNA, along with the verification to exclude high molecular weight DNA contamination, was assessed using the Bioanalyzer 2100 with the High Sensitivity DNA kit (Agilent, Santa Clara, CA, USA). The concentration of the extracted cfDNA was quantified using the Qubit Flex Fluorometer with the dsDNA High Sensitivity kit (Invitrogen, Carlsbad, CA, USA).

### Ethics

The Medical Ethics Committee of the Hospital District of Southwest Finland approved the study protocol (reference No. ETMK 36/180/2012). Each participant gave signed written informed consent before entering the study. The study was performed in accordance with the Declaration of Helsinki.

### Statistical methods

The results are presented as mean ± standard deviation (SD) for variables assumed as normally distributed and median (interquartile range (IQR)) for skewed variables. The normality assumption in continuous variables was evaluated using histograms and Shapiro-Wilk and Kolmogorov-Smirnov tests.

The correlation between cfDNA and biochemical parameters was examined using separate univariate linear regression models and the covariates with a significant association at p < 0.05 were entered separately in multivariable linear regression models adjusted for age, gender and eGFR. Spearman’s rank correlation coefficient was used to examine correlations between two non-normally distributed variables.

The associations in time-based events were examined using separate univariate Cox proportional hazards models and multivariable Cox proportional hazards models adjusted for age, gender, eGFR and coronary artery disease. The date of the baseline cfDNA measurement was used as the starting point for the survival analyses and the analyses were performed for the total follow-up time covering the entire study period as well as the shortened predialysis follow-up time censoring patients at the time of RRT initiation. The association between baseline cfDNA measurement and study outcomes were studied first and in the case of significant associations, the survival analyses were extended to cover associations between the study outcomes and the control measurement of cfDNA and the cfDNA delta ratio. Separate univariate linear regression models (β reported as standardized) were used to analyze the associations between baseline cfDNA and cumulative number of ER visits, hospitalizations and total number of days in hospital care as well as the associations between cfDNA delta ratio and RRT modality at the time of the control measurement. Significant univariate associations were analyzed in multivariable linear regression models adjusted for age, gender and eGFR.

Regarding the analyses exploring the associations between cfDNA delta ratio and RRT modality at the time of the control cfDNA measurement, the patients were divided into three categories (non-dialysis, dialysis and kidney transplantation). Dialysis modalities (hemodialysis or peritoneal dialysis) were not segregated in the analyses. The non-dialysis category included both patients who were still in the predialysis phase and patients on conservative care considered ineligible for RRT or not consenting to initiate RRT.

Independents samples Kruskal-Wallis test and Mann-Whitney U-test with Bonferroni correction were used to compare pairwise or multiple independent groups in non-normally distributed continuous variables. Independent-samples t-tests were used for normally distributed continuous variables and differences in categorical variables were examined using chi-square tests. Wilcoxon signed ranks test was used to compare continuous dependent variables that were not normally distributed.

All statistical analyses were performed using IBM SPSS Statistics 29.0. The significance level was set as two-sided p-value below 0.05 with the explored outcome.

## Results

### Baseline characteristics

Altogether 138 patients with available baseline cfDNA measurement were included in the study and the baseline characteristics are presented in Table 1. The median age was 63 (51–72) years, 51 (37%) were female and median eGFR was 12 (11–15) ml/min/1.73 m^2^. The median baseline cfDNA concentration was 0.197 (0.146–0.312) ng/μl. Median delay between recruitment and the baseline cfDNA sample collection was 84 (54–147) days. Control measurement of cfDNA and thus cfDNA delta ratio was available for 101 patients. The median control cfDNA concentration was 0.266 (0.176–0.467) ng/μl and significantly higher than baseline cfDNA (p < 0.001 for Wilcoxon signed ranks test). The median time between the measurements was 2.7 (2.1–3.7) years and the median for cfDNA delta ratio adjusted for an increment of one year was 1.070 (0.943–1.384) thus equaling a median 7.0% rise in cfDNA per one year of follow-up.

**Table 1:** Baseline characteristics of the study population.

	All subjects (N = 138)	cfDNA < median (N = 69)	cfDNA ≥ median (N = 69)	P-value
**Demographics**				
**Age**	63 (51-72)	60 (46-70)	65 (55-72)	0.024
**Female, N (%)**	51 (37.0%)	24 (34.8%)	27 (39.1%)	0.597
**BMI**	27.9 (24.5-30.7)	27.1 (24.1-30.5)	28.0 (24.7-32.7)	0.273
**Clinical charasteristics**				
**History of smoking, N (%)**	56 (40.6%)	29 (42.0%)	27 (39%)	0.729
**Hypertension, N (%)**	134 (97.1%)	69 (100%)	65 (94.2%)	0.042
**Diabetes, N (%)**	63 (45.7%)	32 (46%)	31 (44.9%)	0.864
**Coronary artery disease, N (%)**	16 (11.6%)	7 (10.1%)	9 (13.0%)	0.595
**Prior myocardial infarction, N (%)**	10 (7.2%)	5 (7.2%)	5 (7.2%)	1.000
**Heart failure, N (%)**	24 (17.4%)	9 (13%)	15 (21.7%)	0.178
**Peripheral artery disease, N (%)**	20 (14.5%)	10 (14.5%)	10 (14.5%)	1.000
**Prior stroke, N (%)**	13 (9.4%)	5 (7.2%)	8 (11.6%)	0.382
**Active malignancy, N (%)**	6 (4.2%)	2 (2.9%)	4 (5.8%)	0.404
**Laboratory measurements**				
**Hemoglobin (g/l)**	115 (±12)	116 (±13)	114 (±11)	0.467
**eGFR (ml/min/1.73m**^**2**^)	12 (11-15)	12 (11-15)	13 (10-15)	0.490
**Urea (mmol/l)**	22.3 (18.5-26.7)	22.3 (18.6-26.8)	22.0 (18.1-26.4)	0.959
**C-reactive protein (mg/l)**	2 (1-4)	2 (1-3)	2 (1-6)	0.119
**ESR (mm/h)**	31 (16-46)	26 (16-37)	35 (19-53)	0.015
**Albumin (g/l)**	35.1 (33.0-37.6)	35.3 (33.4-38.4)	35.1 (31.9-37.5)	0.201
**Calcium ionized (mmol/l)**	1.20 (1.18-1.24)	1.21 (1.18-1.24)	1.20 (1.17-1.24)	0.426
**Phosphate (mmol/l)**	1.44 (1.26-1.67)	1.50 (1.28-1.73)	1.43 (1.25-1.64)	0.161
**PTH (ng/l)**	179 (128-266)	185 (137-300)	172 (105-242)	0.142
**Ferritin (µg/l)**	217 (97-380)	213 (95-370)	225 (103-393)	0.437
**Transferrin saturation (%)**	24.3 (18.6-30.4)	24.4 (19.0-30.8)	24.2 (18.3-29.0)	0.466
**Lactate dehydrogenase (U/l)**	202 (176-230)	193 (171-219)	207 (175-240)	0.045
**Troponin T (ng/l)**	31 (19-52)	32 (18-52)	31 (21-52)	0.860
**ProBNP (ng/l)**	1080 (417-2560)	915 (404-2450)	1260 (463-2725)	0.418
**Cell-free DNA (ng/μl)**	0.197 (0.146-0.312)	0.146 (0.120-0.180)	0.312 (0.246-0.424)	< 0.001

Values are expressed as median (interquartile range) for skewed variables and mean (± standard deviation) for normally distributed variables unless otherwise stated. BMI = body mass index; eGFR = estimated glomerular filtration rate; ESR = erythrocyte sedimentation rate; PTH = parathyroid hormone; proBNP = N-terminal pro-B-type natriuretic peptide.

### Outcomes

Median follow-up time of the study was 6.2 (3.8–7.9) years and altogether 63 (46%) patients died and 35 (25%) were observed with a MACCE, 120 (87%) had an ER visit and 115 (80%) were hospitalized during the study. Only one patient was lost during follow-up. All univariate survival Cox proportional hazard analyses exploring the associations between baseline cfDNA and study outcomes are presented in Table 2.

**Table 2:** The associations between the study outcomes and baseline cfDNA in univariate cox proportional hazards models and multivariable Cox proportional hazards models adjusted for age, gender, eGFR and coronary artery disease at recruitment for the total and predialysis follow-up (median 0.7 (0.3-1.8) years) periods separately.

Outcome	Events, N (%)	Model	HR (95% CI)	P-value
**The total follow-up period**				
**All-cause death**	63 (45.7%)	Univariate Adjusted	0.909 (0.401-2.061) 1.304 (0.431-3.941)	0.819 0.639
**MACCE**	35 (25.4%)	Univariate Adjusted	1.176 (0.549-2.519) 1.584 (0.552-4.544)	0.676 0.393
**ER visit**	120 (87.0%)	Univariate Adjusted	0.748 (0.335-1.667) 0.896 (0.411-1.953)	0.477 0.781
**Hospitalization**	115 (83.3%)	Univariate Adjusted	0.696 (0.338-1.433) 0.691 (0.330-1.448)	0.325 0.328
**Hospitalization due to infection**	98 (71.0%)	Univariate Adjusted	1.084 (0.681-1.727) 1.154 (0.725-1.836)	0.734 0.545
**Hospitalization due to CV cause**	59 (42.8%)	Univariate Adjusted	0.832 (0.342-2.028) 1.001 (0.328-3.059)	0.686 0.998
**Incident malignancy (N = 132)** ^**a**^	19 (14.4%)	Univariate Adjusted	0.877 (0.195-3.952) 1.652 (0.202-13.528)	0.864 0.640
**Predialysis follow-up period (censoring at RRT initiation)**				
**All-cause death**	10 (7.2%)	Univariate Adjusted	2.153 (0.040-114.804) 1.596 (0.012-208.382)	0.705 0.851
**MACCE**	10 (7.2%)	Univariate Adjusted	0.985 (0.052-18.627) 1.410 (0.115-17.292)	0.992 0.788
**ER visit**	49 (35.5%)	Univariate Adjusted	1.741 (0.905-3.347) 1.858 (0.976-3.538)	0.097 0.059
**Hospitalization**	101 (73.2%)	Univariate Adjusted	0.616 (0.196-1.933) 0.806 (0.335-1.938)	0.406 0.630
**Hospitalization due to infection**	23 (16.7%)	Univariate Adjusted	1.035 (0.158-6.793) 1.348 (0.224-8.104)	0.971 0.744
**Hospitalization due to CV cause**	19 (13.8%)	Univariate Adjusted	0.575 (0.069-4.808) 0.684 (0.041-11.390)	0.609 0.791
**Incident malignancy (N = 132)** ^**a**^	7 (5.3%)	Univariate Adjusted	2.169 (0.275-17.084) 2.654 (0.152-46.441)	0.462 0.504

eGFR = estimated glomerular filtration rate; cfDNA = cell-free DNA; HR = hazard ratio; CI = confidence interval; MACCE = major cardiovascular or cerebrovascular event; ER = emergency room; CV = cardiovascular; RRT = renal replacement therapy. ^a^Patients with baseline malignancy excluded.

The cumulative median number of ER visits was 6 (3−10) and cumulative median number of hospitalizations and total days under hospital care were 7 (4−12) and 35 (19−69) days respectively. In separate univariate linear regression analyses, baseline cfDNA concentration was not associated with the cumulative number of ER visits (β 0.104, p = 0.226) or hospitalizations (β 0.117, p = 0.172) or with the cumulative number of days under hospital care (β 0.045, p = 0.600). Furthermore, no association was observed between baseline cfDNA concentration and cumulative ER visits or hospitalizations when only ER visits or hospitalizations due to infection (β 0.094, p = 0.271 and β 0.030, p = 0.728 for ER visits and hospitalizations respectively) or cardiovascular causes (β −0.119, p = 0.163 and β −0.042, p = 0.627 for ER visits and hospitalizations respectively) were included in separate univariate linear regression analyses (S1 Table).

Of the 132 patients with no baseline malignancy, 19 (14%) were observed with an incident malignancy during follow-up and incident malignancy was not associated with baseline cfDNA concentration (univariate Cox proportional hazards analysis HR 0.877, CI 95% 0.2195–3.952, p = 0.864).

### Associations between baseline characteristics and baseline cfDNA

None of the baseline characteristics regarding patient demographics or disease history were associated with baseline cfDNA in univariate linear regression models. Measurements of hemoglobin, plasma urea, parathyroid hormone, ferritin and lactate dehydrogenase were associated with baseline cfDNA in separate univariate linear regression analyses. All of these associations remained independently associated with baseline cfDNA in separate multivariable linear regression models adjusted for age, gender and eGFR (Table 3, S1 Table).

**Table 3:** Associations between baseline laboratory measurements and baseline cfDNA ratio in multivariable linear regression analyses adjusted for age, gender and baseline eGFR.

	Baseline cfDNA (N = 138)		
Covariate	β	95% CI	P-value
**Hemoglobin**	−0.005	−0.010 - −0.001	0.024
**Urea**	0.009	0.001 - 0.017	0.029
**CRP**	0.002	−0.004 - 0.008	0.608
**ESR**	0.000	−0.002-0.003	0.950
**LDH**	0.003	0.002 - 0.003	**< 0.001**
**Albumin**	0.009	−0.005-0.023	0.206
**Ferritin** ^**a**^	0.368	0.321 - 0.414	< 0.001
**TSAT**	−0.003	−0.009 - −0.003	0.278
**Ionized calcium**	−0.370	−1.283 - 0.543	0.424
**Phosphate**	0.007	−0.201 - 0.214	0.950
**PTH**	0.001	0.000 - 0.001	0.004
**Troponin T**	0.001	0.000-0.003	0.131
**ProBNP**	0.000	0.000-0.000	0.786

Multivariable linear regression model adjusted for age, gender and eGFR. β reported as unstandardized. cfDNA = cell-free DNA; CI = confidence interval; eGFR = estimated glomerular filtration rate; CRP = C-reactive protein; ESR = erythrocyte sedimentation rate; LDH = lactate dehydrogenase; TSAT = transferrin saturation; PTH = parathyroid hormone; proBNP = N-terminal pro-B-type natriuretic peptide. ^a^ per increment of 1000 µg/l.

### The associations between cfDNA delta ratio and RRT modality at the time of the control measurement

Altogether 45 patients were receiving maintenance dialysis, 35 had received a kidney transplant and 21 were on conservative care at the time of the cfDNA control measurement. The baseline cfDNA concentrations were similar between the groups (p = 0.135 for Kruskal-Wallis test). The predialysis vintage (the time from recruitment to RRT initiation) was 145 (76–393) days in patients who were undergoing dialysis at the time of the control measurement and 192 (113–357) days in kidney transplant recipients. The dialysis vintages were 624 (411–1231) and 387 (179–598) days in these patients, respectively. The transplant vintage at the time of the control measurement was 396 (188–806) days. All kidney transplant recipients had received dialysis prior to the transplantation.

**Fig 2:**
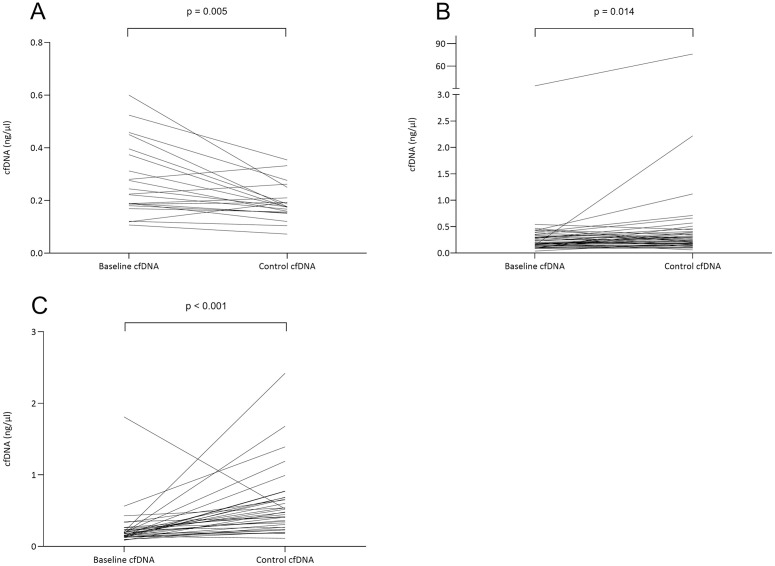
Individual trajectories of baseline and control cfDNA concentrations in patients not undergoing dialysis (A), undergoing maintenance dialysis (B) and kidney transplant recipients (C) at the time of the control measurement. The difference between the two time points assessed using the Wilcoxon signed ranks test within each group.

**Fig 3:**
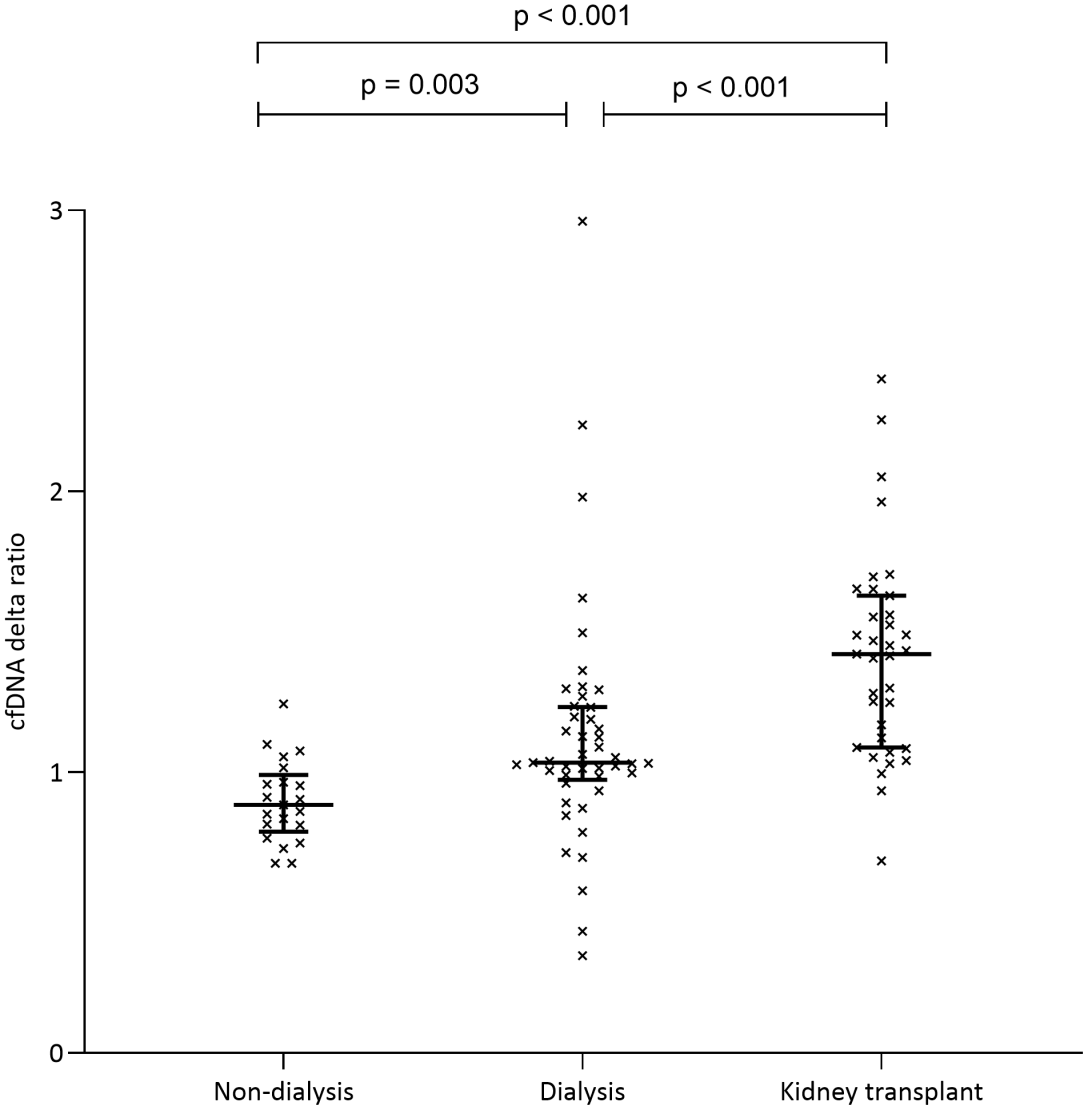
cfDNA delta ratio by treatment modality at the time of the cfDNA control measurement. Median values (IQR) for non-dialysis 0.884 (0.788–0.990), dialysis 1.034 (0.973–1.233) and kidney transplant 1.421 (1.087–1.628) groups. Significant differences in pairwise comparisons between groups “non-dialysis” and “dialysis” (p = 0.003), “non-dialysis” and “kidney transplant” (p < 0.001) and “dialysis” and “kidney transplant” (p < 0.001) using Mann-Whitney U-test with Bonferroni adjustment.

In groupwise analyses, the differences between baseline and control cfDNA measurements in patients not receiving dialysis, undergoing maintenance dialysis or kidney transplant recipients at the time of the control measurement were statistically significant (p < 0.02 for all comparisons). The evolution of cfDNA between the baseline and control measurements is illustrated in Fig 2. Patients who were undergoing dialysis had significantly higher cfDNA delta ratio compared to those who were not, and patients who had received a kidney transplant had significantly higher cfDNA delta ratio compared to dialysis patients and those receiving conservative care (Fig 3).

**Fig 4:**
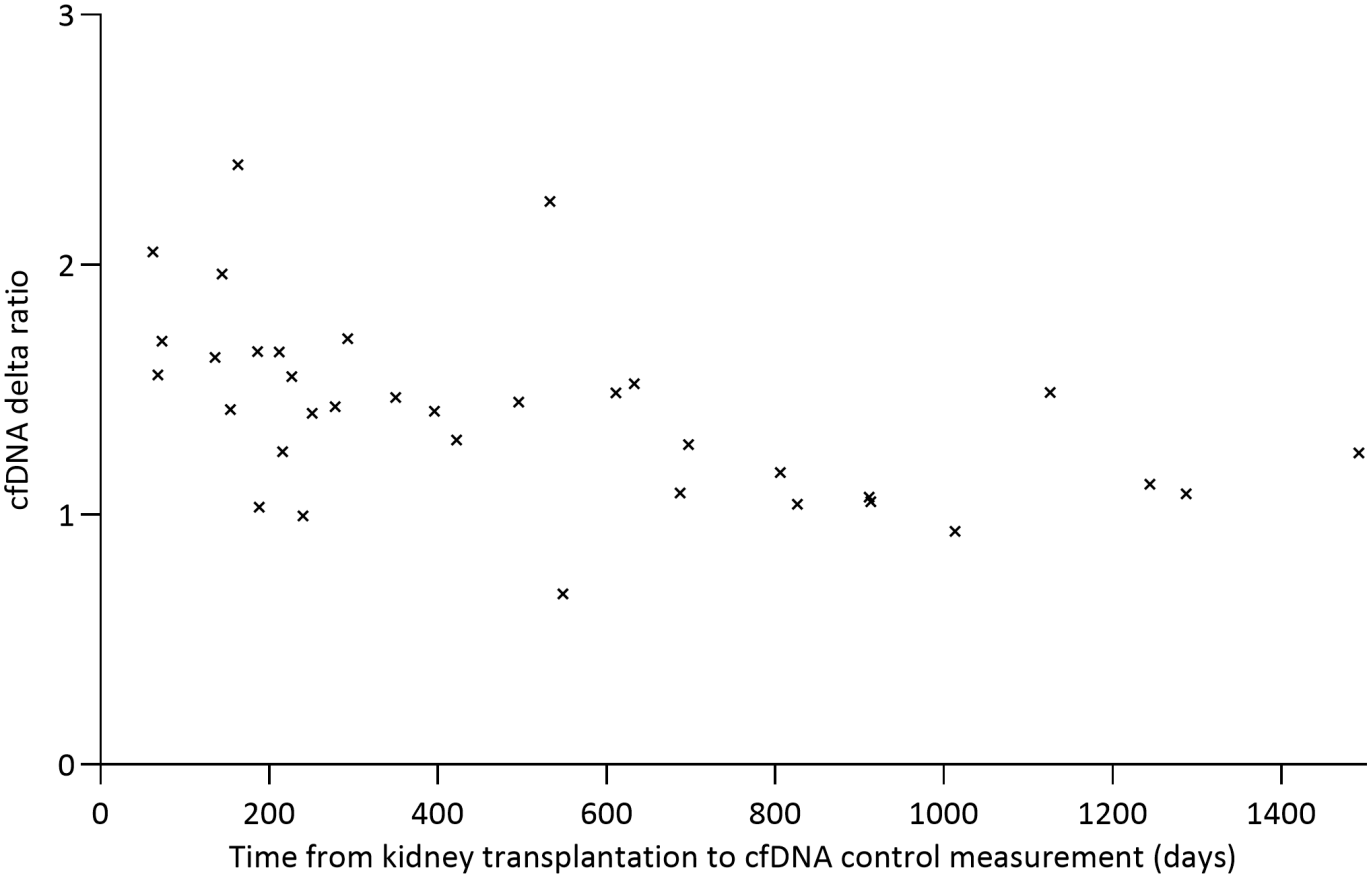
Correlation between kidney transplant vintage and cfDNA delta ratio in kidney transplant recipients. Spearman’s rank correlation coefficient −0.592, p < 0.001.

In patients undergoing maintenance dialysis at the time of the control cfDNA measurement, cfDNA delta ratio was not associated with dialysis vintage in a univariate linear regression model (β −0.107, p = 0.484). However, kidney transplant recipients’ cfDNA delta ratio was associated with transplant vintage in a univariate linear regression model (β −0.493, p = 0.003) (Fig 4) and in a multivariable linear regression model (β −0.586, p = 0.002) adjusted for age, gender, eGFR and both predialysis and dialysis vintage (S1 Table).

## Discussion

The present work is the first to study the associations between cfDNA and adverse outcomes as well as the evolution of cfDNA between two time points in non-dialysis CKD stage 4–5 patients transitioning to different modalities of RRT or continuing on conservative care. Plasma cfDNA was not associated with mortality or any of the adverse outcomes. Furthermore, none of the baseline characteristics and only few baseline laboratory measurements were associated with baseline cfDNA. However, the change from the baseline to the control measurement of cfDNA differed according to the modality of care at the time of the control measurement.

Inflammation is an inherent and clinically relevant feature in CKD especially at the more severe stages [17] and known to be associated with adverse outcomes and mortality [18]. Elevated CRP, a biomarker for inflammation, has been shown to be associated with mortality in advanced CKD [5] and a correlation between cfDNA and CRP has been observed both in the general population and in patients with CKD [19–21]. Consequently, cfDNA, a more novel and potentially more sensitive biomarker for inflammation and tissue damage, has been the focus of fervent research to establish a more advanced clinical tool for risk stratification in CKD [14,21–23]. Previous literature suggests that cfDNA is associated with the risk of mortality and cardiovascular disease burden in both the general population and elderly individuals. [15,20]. Research on cfDNA in CKD has however, been scarce, and all prior data are limited to patients on maintenance dialysis. In dialysis patients, cfDNA, especially post-hemodialysis measurement of cfDNA, has been associated with mortality. [21–23]. On the contrary, we observed no association between baseline cfDNA and mortality or incident MACCEs in CKD stage 4–5 patients not receiving dialysis at baseline. Baseline cfDNA was negatively associated with hemoglobin and positively with plasma urea, parathyroid hormone, ferritin and lactate dehydrogenase possibly reflecting the complex network between cfDNA and inflammation despite the fact that baseline cfDNA measurement was not associated with CRP. In line with our data, a positive association with ferritin, an acute-phase protein, and a negative association with hemoglobin and cfDNA have been observed in prior literature on hemodialysis patients while a negative association with PTH has been observed in another in contrast to our findings [23]. Ultimately, the reason for the inconsistencies between our findings and prior literature is not clear but it is possible that cfDNA may be a more subtle marker for tissue damage whose signal is partly “drowned” in the presence of the more robust inflammation associated with advanced CKD. This point is reinforced by the moderately elevated median erythrocyte sedimentation rate in our cohort – a typical phenomenon in patients with CKD stage 4–5 [24]. Further clarification is needed to determine whether cfDNA has clinical potential in the risk assessment of CKD stage 4–5 patients in contemporary clinical nephrology.

Kidney transplantation has been shown to decrease the level of inflammatory biomarkers [25] but data on the effect on cfDNA levels are scarce. A study by Leotta et al in 2023 examined cfDNA prior to and two years after kidney transplantation and found no difference in the concentrations of cfDNA. [26]. We, however, observed a statistical difference between the baseline and control cfDNA measurements in the patients that had received a kidney transplant prior to the control measurement. This cfDNA delta ratio was numerically higher in kidney transplant recipients and significantly different from patients receiving conservative care or dialysis at the time of the control cfDNA measurement thus suggesting a higher degree of alteration in the inflammatory landscape among these patients. The effect of kidney transplantation on cfDNA and the evolution of cfDNA or any inflammation biomarker in this setting is poorly understood, as the overlapping and possibly opposing effects of immunosuppression, resolving kidney function and the effect of kidney graft on inflammation are difficult to distinguish from one another. We observed a negative correlation between the cfDNA delta ratio and kidney transplant vintage (that is, the difference between the cfDNA measurements became less pronounced with increasing delay between transplantation and the control cfDNA measurement) in transplant recipients. It is possible that the higher control cfDNA concentrations in patients with a control measurement shortly after kidney transplantation may be associated with the early post-transplantation phase (recovery from the operation, healing tissue damage and adjustment of the immune system to the presence of the kidney graft and high doses of immunosuppressive medications) and lower cfDNA concentrations observed in patients with longer delays between transplantation and the control measurement are associated with the more stable condition of the graft and lower intensity of immunosuppression. Concordantly, the difference in timing of the control cfDNA measurement may explain the difference in the results between our work and prior literature as the median kidney transplant vintage in our study was approximately a year compared to two years in the study by Leotta et al [26]. Nevertheless, inflammatory status seems to be associated with mortality in kidney transplant recipients [27] and a similar connection with cfDNA is possible. The current clinical application of cfDNA in nephrology, however, is limited to the diagnosis and surveillance of allograft rejection. This implementation of cfDNA as a biomarker in kidney transplant rejection has relied on the detection of donor-derived cfDNA in the plasma of the recipient and not the total cfDNA as measured in our study [28].

According to prior data, hemodialysis patients have been observed with higher cfDNA concentrations than healthy controls [21] and cfDNA concentrations have been observed to rise with dialysis vintage in repeated measurements [22]. It has also been shown that hemodialysis itself increases cfDNA concentration regardless of the type of dialysis membrane employed, possibly due to leukocyte apoptosis. [29]. Thus, it was not a surprise that the median cfDNA concentrations between the baseline and control measurement increased in individuals undergoing maintenance dialysis. However, there was variance in the change between the two measurements (Fig 2) and dialysis vintage was not associated with the cfDNA delta ratio in our study. This finding is somewhat unexpected as an increase in cfDNA levels might have been anticipated with the progression of kidney dysfunction and related inflammation. However, mortality before the initiation of RRT in high risk patients and, on the other hand, the slower overall progression of CKD in patients with more favorable prognosis may have created selection bias in our cohort. It is also possible that the improved clearance of uremic inflammatory toxins associated with dialysis and the exposure to different dialysis machinery and fluids depending on the modality of dialysis may have opposing effects on inflammation and explain the variance in the cfDNA delta ratio in patients that initiated dialysis prior to the control cfDNA measurement.

Almost 9 out of 10 patients in our study cohort had an ER visit and 4 out of 5 were hospitalized during the follow-up. These numbers are not unexpected given the substantial burden of comorbidities in the study patients and long follow-up period. To our knowledge, no previous data have been published on the associations between cfDNA and hospitalizations or ER visits, regardless of kidney function. No association between cfDNA and ER visits or hospitalizations was observed in our study. This is relatively surprising as ER visits and hospitalizations are associated with a variety of adverse events and health emergencies such as traumatic injuries, infections and cardiovascular events all of which are known to be associated with cfDNA [11–13]. Moreover, no link between cfDNA and hospitalization due to infection or cardiovascular causes in separate analyses was observed. The very high rate of ER visits and hospitalizations may have complicated the statistical analyses but substantial use of health care services is typical for patients with CKD stage 4–5.

cfDNA-based tests have also been developed to serve as a screening tool for early detection of malignancies as changes in cfDNA may precede the symptoms and clinical onset of cancer [30]. Although the association between rising concentrations of cfDNA and malignancies was observed almost 50 years ago, no data on the link between cancer and cfDNA in the presence of CKD exist [31]. In our study, every tenth patient was observed with an incident malignancy during follow-up but no association between baseline cfDNA and the risk of incident malignancy was observed. Altogether the clinical potential of an unselective and sensitive biomarker such as the total concentration of cfDNA for the prediction of incident malignancies, adverse outcomes or mortality in patients with severe kidney dysfunction and associated inflammation may not be optimal, especially so in those undergoing dialysis or receiving a kidney transplant. The evolution of inflammation in CKD and associated modalities of care are complex and still incompletely understood making the implementation of novel inflammatory biomarkers like cfDNA difficult in the field of clinical nephrology.

This study has the inherent limitations of an observational study and causality cannot be established from our findings. Additionally, the sample size was relatively small, not all patients had cfDNA measurements available, and the study represents observations from a single center with rather homogenous ethnicity, which may limit the generalizability of our results. cfDNA stability is highly sensitive to preanalytical conditions. EDTA tubes minimize DNase-mediated degradation compared with heparin and serum tubes when processed promptly [32]. To mitigate these factors, we followed standardized laboratory protocols, used a commercial cfDNA isolation kit, and confirmed fragment size profiles with a Bioanalyzer. This allowed us to detect and exclude samples with high-molecular-weight genomic DNA contamination, ensuring reliable cfDNA measurements throughout the study. Although cfDNA is not yet routinely used in contemporary clinical nephrology, all the previous four clinical studies to evaluate cfDNA in patients with advanced chronic kidney disease had used fluorometric assays in their analyses [21–23,26]. The variation in the timing of the cfDNA control measurement was not optimal and necessitated the expression of the delta as an increment of one year of follow-up to account for the variance in the delay between the cfDNA samples. Moreover, our protocol only included the baseline measurement and one control time point despite additional control cfDNA measurements, particularly at RRT initiation, would have been more optimal. However, the follow-up time was long compared to previous studies [21–23] and the event rate of the studied outcomes was high. Furthermore, the quality of our manually collected outcome data is high, and the follow-up comprehensive eliminating uncertainties associated with registry studies relying on electronic data searches. The laboratory sample collection was done concurrently with clinical visits and/or before in-center hemodialysis sessions in terms of the control cfDNA measurement. Thus, coincident episodes of infections may have skewed our measurements in some patients as cfDNA concentrations tend to rise at the time of acute illness. However, we believe the confounding effect of infections to be scarce and somewhat expected in a real-world study. There was some heterogeneity among the patients receiving different modalities of care at the time of the control cfDNA measurement as the patient group on maintenance dialysis included patients eligible and ineligible for kidney transplantation and patients not on RRT included those with a selected conservative/palliative treatment strategy as well as predialysis patients not transitioned to RRT yet. Furthermore, patients receiving hemodialysis or peritoneal dialysis were not segregated in this study due to the sample size. Despite these limitations, our study is the first to provide data on the evolution of cfDNA in predialysis CKD stage 4–5 patients transitioning to different modalities of RRT in a real-world setting.

In conclusion, our study is the first to explore the link between cfDNA and mortality and adverse outcomes and the evolution of cfDNA in CKD stage 4–5 patients not undergoing dialysis at baseline transitioning to different modalities of RRT or continuing on conservative care. No association between cfDNA and the studied adverse outcomes was observed.

## Supporting information

S1 Table Full results of the linear regression analyses performed. β reported as unstandardized. cfDNA = cell-free DNA; CI = confidence interval; eGFR = estimated glomerular filtration rate; CRP = C-reactive protein; ESR = erythrocyte sedimentation rate; LDH = lactate dehydrogenase; TSAT = transferrin saturation; PTH = parathyroid hormone; TnT = Troponin T; proBNP = N-terminal pro-B-type natriuretic peptide; ER = emergency room; CV = cardiovascular; RRT = renal replacement therapy; ^a^ per increment of 1000 µg/l.
(XLSX)
